# Implementation and scale up of population physical activity interventions for clinical and community settings: the PRACTIS guide

**DOI:** 10.1186/s12966-018-0678-0

**Published:** 2018-06-08

**Authors:** Harriet Koorts, Elizabeth Eakin, Paul Estabrooks, Anna Timperio, Jo Salmon, Adrian Bauman

**Affiliations:** 1 Institute for Physical Activity and Nutrition (IPAN), School of Exercise and Nutrition Sciences, Deakin University, Geelong, Australia; 2 School of Public Health, Cancer Prevention Research Centre, The University of Queensland, Herston, QLD Australia; 3 Department of Health Promotion, College of Public Health, University of Nebraska Medical Center, 984365 Nebraska Medical Center, Omaha, NE USA; 4 School of Public Health, University of Sydney, Sydney, NSW Australia

**Keywords:** Implementation, Physical activity, Translation, Dissemination, Health behaviour, Public health

## Abstract

**Background:**

Few efficacious physical activity interventions are successfully translated and sustained in practice. We propose a practical guide for researchers to increase the likelihood of successful implementation and scale up of physical activity interventions in practice contexts. The guide is based on two principles: (i) differences between the research and practice context can be addressed during intervention development and implementation planning by focusing on system, delivery personnel, and intervention characteristics; and (ii) early planning for implementation barriers and facilitators can improve subsequent translation into practice.

**Methods:**

From the published literature, we identified evidence of strategies to improve research-practice translation, along with narrative descriptions of different approaches to addressing translational challenges. These, along with constructs taken from widely cited implementation outcome, process, and mechanistic models were collated and inform the guide.

**Results:**

The resultant PRACTIS guide (**PRACT**ical planning for **I**mplementation and **S**cale-up) comprised the following four iterative steps: *Step 1*) Characterize the parameters of the implementation setting; *Step 2*) Identify and engage key stakeholders across multiple levels within the delivery system(s); *Step 3*) Identify contextual barriers and facilitators to implementation, and; *Step 4*) Address potential barriers to effective implementation.

**Conclusions:**

A lack of practical guidance for researchers on how to effectively plan implementation and scale up of physical activity interventions prevents us moving quickly from evidence to action. We recommend that intervention development and adaptation for broad and sustained implementation be prioritized early in intervention planning and include active engagement from delivery organizations and stakeholders. The PRACTIS guide is also relevant for clinical and public health researchers in other areas of prevention.

**Electronic supplementary material:**

The online version of this article (10.1186/s12966-018-0678-0) contains supplementary material, which is available to authorized users.

## Background

The past decade has seen a significant rise in the number of efficacious interventions to promote physical activity [1]. Although promising, only a minority of these interventions move from research into practice, and those that do provide limited information on sustainability or institutionalization within routine practice [2]. The movement of research to practice settings is a dynamic process that includes dissemination, implementation, and scale-up. The National Institutes of Health (NIH) define dissemination as the targeted distribution of information and intervention materials to a specific public health or clinical practice audience, with the intent to spread knowledge and the associated evidence-based interventions [3]. Implementation is the use of strategies to adopt and integrate evidence-based health interventions and change practice patterns within specific settings [3]. Scale-up is described as replicating and extending the reach of an intervention into other localities, cities, or regions [2]. Scalability describes an intervention’s potential to be delivered to an increasing number of participants or through an increasing number of settings, while retaining effectiveness [4].

There are some clear examples of dissemination [5], implementation [6], and scale-up of physical activity interventions [7, 8]. However, across three decades of physical activity intervention research, the majority of publications have been efficacy/effectiveness trials and only 3% comprised dissemination studies [4]. This continued lack of evidence for the successful institutionalisation of physical activity interventions in real-world settings, combined with unacceptably high levels of physical inactivity worldwide [9, 10], makes addressing the research-to-practice gap a significant public health priority.

Implementing and sustaining physical activity interventions in practice contexts is challenging [11, 12]. These challenges are grounded in the social-ecological differences between testing a physical activity intervention within an efficacy trial under optimal conditions to dissemination, implementation, and scale-up trials where multilevel infrastructure, resources, values, and characteristics of participants are much more variable [13–15]. In public health and health promotion research, dissemination is often an afterthought in the program life cycle, or conceptualized as a separate process that occurs at some nonspecific time after efficacy trials are completed [16]. Features of the implementation context which can influence the impact of an intervention (i.e. delivery capacity of implementers and organizations [17]), may also only be addressed when an intervention is considered ready for dissemination. As a result, when efficacious interventions are implemented in real-world settings, they can either fail to be adopted or report lower effect sizes, and are less likely to be sustained over time [5, 18].

There have been calls for the research community to shift the focus from small scale tightly controlled interventions to evaluating those capable of dissemination and translation [2]. Pragmatic trials [19], hybrid effectiveness-implementation trials [20] and participatory research approaches [21–23] are examples of study designs more suited to community and clinical-relevant research. These approaches engage key stakeholders, prioritize implementation outcomes and assess the generalizability of intervention effects. In addition, there have been calls to improve the translation of research into practice by considering a broader set of metrics, such as generalisability and sustainability, and to increase the adoption of theory-driven implementation research. For example, systems science methods (e.g. social network analysis, agent-based modelling and system dynamics) can elucidate the effects of dissemination and implementation barriers and facilitators on intervention outcomes [24]. Measurement of implementation outcomes, such as intervention feasibility and implementation cost, can be used as a proximal indicator of implementation processes, success, and sustainability [25].

There has been an increased recognition of the importance of theory-driven approaches to enhance dissemination and implementation research [26]. Extending beyond Roger’s Diffusion of Innovations Theory [27], which underpins a large body of dissemination research, there are currently over 60 published theories, models and frameworks [28]. Although methodological advances to support dissemination, implementation and scale-up trials are promising, theories, models and frameworks do not provide guidance on how best to initiate contextually-relevant processes to engage and work with typical community or clinical organizations. There is a gap in the current literature which explicitly describes ‘*how to*’ enact strategies to successfully translate research into practice.

The ‘how to’ guide proposed here does not purport to be a new framework or model. Rather, it draws on existing models and frameworks to address *how* to plan for implementation and scale-up during intervention development, testing and ongoing adaptation. We propose that prioritising factors relevant to dissemination, implementation, and scale-up early within the research process, will enable potential barriers to be addressed and their impact measured; although this remains to be tested and is beyond the scope of this paper. The PRACTIS (**PRACT**ical planning for **I**mplementation and **S**cale-up) guide outlines a structure for researchers and stakeholders, with varying levels of implementation experience and expertise, to navigate the complex considerations and decision-making processes involved in translating evidence-based interventions into practice. The purpose is to increase the likelihood that physical activity interventions can be implemented at scale and sustained in practice contexts. The challenges associated with intervention implementation and scale-up are relevant across all areas of public health prevention. For the purposes of this paper, therefore, we refer to physical activity intervention research to discuss these issues and illustrate operationalization of the PRACTIS guide.

## Methods

The guide was developed based on two bodies of relevant literature addressing strategies to improve research-practice translation, and constructs associated with effective implementation from widely cited implementation outcome, process, and mechanistic models.

To identify processes to improve research-practice translation, we refer to literature outlining strategies used to enhance implementation of evidence-based practices [29, 30], and the challenges of making research findings relevant to community and clinical systems [12, 31–33]. Common critiques include lack of representativeness of participants and intervention implementation staff, and a focus on achieving the largest effect possible, without a focus on the financial, resource, and human capital costs or even a consideration of the most likely system that would ultimately implement a physical activity intervention. Specifically, when planning an intervention, it is recommended to consider strategies to address participant reach and representativeness, organizational staff and system adoption, delivery ease and quality of implementation, and intervention sustainability [34]. Our summary of this literature encouraging an expansion of outcome assessment within dissemination, implementation, and scale-up research is included in the guide. It informed the development of a comprehensive checklist to support researcher and stakeholder planning that addresses strategies to improve implementation, dissemination and scale-up, and tools to aid the decision making processes used to develop partnerships with systems that will ultimately apply research findings within practice.

To identify constructs associated with effective implementation, we referred to a second body of literature summarizing implementation theories, models and frameworks [26, 28]. Frameworks were chosen for inclusion in the PRACTIS guide based on the following criteria: (i) a typology of constructs associated with effective dissemination, implementation, and/or scale-up; (ii) specifying causal multi-level associations with implementation effectiveness; (iii) developed based on either empirical results or unifying constructs from existing conceptual frameworks/theories; (iv) previous application in physical activity and preventive health research and; (v) applicability to a variety of implementation settings. Three widely cited explanatory frameworks were identified: the Interactive Systems Framework (ISF) [17], the ecological framework for effective implementation [14], and the Consolidated Framework for Implementation Research (CFIR) [35].

Constructs within each framework were individually tabulated and grouped based on five higher order domains described within the frameworks (implementer characteristics; organizational characteristics; intervention characteristics; community characteristics and; process of implementation). We compared and contrasted the constructs and propositions from each of these frameworks, and identified overlapping constructs based on a shared definition or meaning (i.e. ‘skills’ in the ISF and ‘skill proficiency’ in Durlak and Dupre’s ecological framework), and a comparable level of impact (i.e. organizational or community level). A total of 85 individual constructs existed across the three frameworks, which resulted in 72 overlapping ‘common’ constructs, to produce a compilation of multi-level barriers to implementation (see Additional file 1 which presents the framework constructs). As the five higher order domains from these frameworks span all levels of the social-ecological model, they were used as areas of emphasis throughout our proposed guide.

A draft of the guide was piloted among physical activity and nutrition experts (*n* = 9) during an Implementation Science workshop held at an Australian University in October 2017. Registration was voluntarily and there were no costs to participate. Feedback during this workshop led to revisions to the explanatory text within the guide, to improve use ability and application of the guide in practice.

### The proposed PRACTIS guide

The guide is based on the following underlying principles. First, multi-level differences that exist between the research (intervention testing) and practice (intervention implementation) context can be best addressed during early intervention planning that engages stakeholders. Second, early anticipation and planning for barriers to intervention implementation, typical in community or clinical settings, can improve the translation of evidence to practice.

**Fig. 1:**
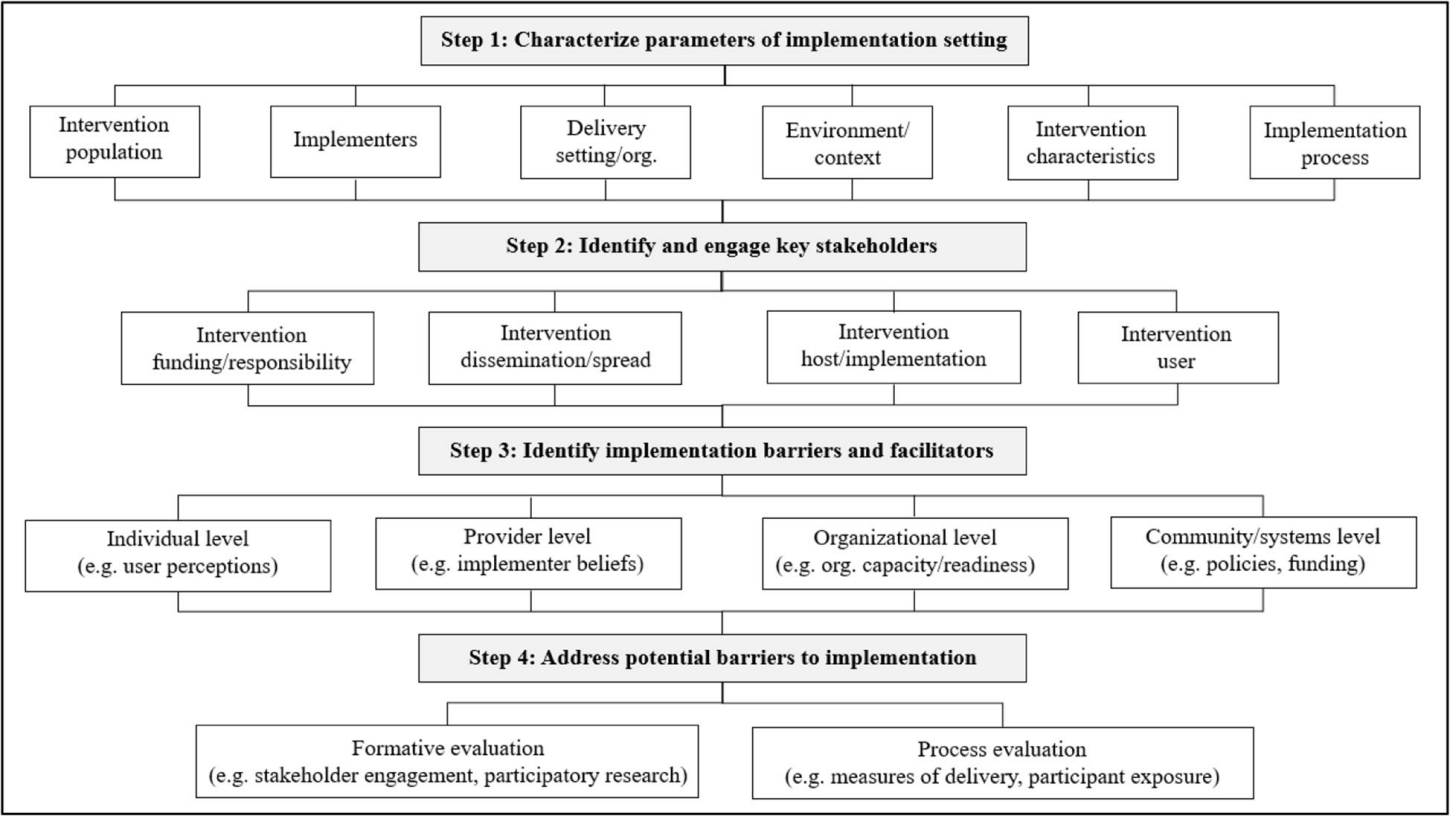
PRACTIS guide steps

The guide describes a series of steps outlining how best to plan the dissemination, implementation, and scale-up of physical activity interventions for clinical and public health settings: ***Step 1***) Characterize the parameters of the implementation setting; ***Step 2***) Identify and engage key stakeholders across multiple levels within the delivery system(s); ***Step 3*)** Identify contextual barriers and facilitators to implementation, and; ***Step 4***) Address potential barriers to effective implementation. Characteristics of the potential implementation context feature as the foremost component of the guide. The ‘implementation context’ refers to the physical, social and cultural environment where the physical activity intervention would be integrated. The implementation context also includes who, what and how the intervention would be delivered if research funding has ceased. Characteristics of the implementation context (*Step 1*) underpin all decisions and strategies to improve implementation planning and intervention testing (*Steps 2-4*). Figure 1 presents an overarching process flowchart of the steps required.

The process begins by engaging key stakeholders to consider: the population who will receive or deliver the intervention; the place where the intervention will be implemented; the intervention and implementation process; the provisions needed for implementation; and the principles underpinning scale up. Optimally, iterative evaluation is used to refine implementation strategies for dissemination, for sustained implementation, and to assess outcomes when scale-up is complete.

Outcomes from *Steps 1- 4* are likely to contribute to more effective strategies for intervention implementation, and also to assessments of implementation-related outcomes (e.g. reach, delivery quality, acceptability, compatibility, costs). Each *Step* considers multi-level influences on implementation of physical activity interventions in practice settings. Common constructs identified from within key implementation frameworks inform potential barriers in *Step 3*. Formative and process evaluation approaches [36, 37], including intervention testing methodologies [12, 31, 34], inform strategies to address potential barriers to effective implementation in *Step 4*.

We present these steps as a linear process of repeated cycles, but acknowledge that steps are not mutually exclusive and the process is iterative, thus will reflect learnings from systematic implementation efforts. For example, identifying barriers in *Step 3* may lead to revisiting how the parameters of the implementation setting are characterized. The *Steps* may occur sequentially, include overlapping activities, and/or in a different order depending on the complexity of the intervention and setting. The *Steps* should be followed as contextually appropriate.

### Step 1: Characterize parameters of the implementation setting

*Step 1* describes features of the proposed implementation setting, pinpointing gaps in the planning process. Early familiarization with characteristics of the real-world implementation context promotes planning and accountability for future implementation, and may enhance implementation efforts. Table 1 presents a 15-item checklist containing 38 questions to guide researcher and stakeholder familiarisation with the potential implementation setting. Researchers *and* stakeholders can complete the checklist independently, to enable differences in perspectives and expectations regarding implementation to be highlighted and addressed. Questions within this checklist can also be used to guide researcher-stakeholder discussions on target areas of importance (*Step 2*). As disparities exist in physical activity participation [38], special consideration is given across the checklist to reflect on high need populations that may be more at risk of physical inactivity due to particular disparities. The process within this guide is iterative and therefore some checklist items may only be answerable after stakeholder consultation or intervention piloting, for example. The checklist should be used to identify gaps or areas for further exploration throughout the implementation and scale up process as new information emerges.

**Table 1:** Checklist considerations when characterizing implementation setting parameters (Step 1)

Level	Characteristics	Process of intervention adoption and delivery	Sustainability
Intervention population (Individual level)	1. Who will access the intervention? What is the size of the target population? Are there participation eligibility criteria (i.e. age)? Are there subgroups that experience disparities in physical activity?	2. How will the target population access/be recruited into the intervention? What will motivate or incentivize them to take part? How will you ensure equity of access for disadvantaged subgroups?	3. How will retention be supported and monitored? How will you ensure those who may be at higher risk of attrition will be retained and how will this be monitored?
Implementers (Provider level)	4. Who will deliver the intervention? How many implementers will be required? Are there eligibility criteria to deliver the intervention (i.e. level of skill, knowledge, education)?	5. How will implementers be identified/engaged and trained? What will motivate or incentivize them to implement the intervention? How will you facilitate engagement with disadvantaged groups?	6. How will implementers be supported (i.e. ongoing training, performance feedback, champions) to sustain intervention fidelity and delivery? How will you prepare for sustainability in lower-resourced settings?
Delivery setting/org. (Organizational level)	7. What is the target delivery setting(s) (i.e. setting, size) and are there eligibility criteria for adoption (i.e. possess certain resources)? How will you engage settings that provide services to disadvantaged subgroups?	8. How will target delivery settings be identified and be made aware of the intervention? What will motivate or incentivize the setting to adopt and implement the intervention?	9. Who will take ownership of the intervention and how will adoption, delivery, impact, and sustainability be monitored? How will start-up and ongoing costs be considered when planning for sustainability and implementation at scale?
Environment/ context (Community/ systems level)	10. What are the key characteristics of the target community (i.e. built environment infrastructure, low-high income)? How will you engage communities with disadvantaged subgroups?	11. How will characteristics of the community (i.e. funding and political climate, readiness for implementation) influence dissemination, implementation and scale-up? How will community accountability for implementation be generated and assessed?	12. Who at the community/systems level will be responsible for the intervention? Are there individual or organizational champions for intervention implementation that could help to plan for sustainability?
Intervention factors: (All levels)	13. What is the intervention design (i.e. strategies, underlying principles, delivery format, duration, resources required)? What are the core and adaptable elements (i.e. flexibility)? Which elements may/may not be scalable? How simple/complex is the design and what relative advantage does the intervention provide?	14. How will the intervention and plans for implementation, be developed so they align with organizational missions, values and infrastructure (i.e. size, resource availability)? How will the intervention integrate into existing individual and organizational practices (i.e. setting compatibility)?	15. How will the intervention and associated costs and resources for delivery (i.e. materials) be sustainably funded? How will intervention implementation processes (i.e. setting/staff training) be integrated into organizational policies and job descriptions? How will implementation capacity be developed and sustained at scale?

Checklist items correspond to key issues relating to intervention adoption, delivery and sustainability in practice, potential scalability of the intervention and implementation process, and important multi-level contextual characteristics. Checklist responses contribute to describing: who/how many people the intervention will reach and which individuals will be involved/required for effective implementation (i.e. People); what settings/organizations will be involved/required (i.e. Place); how the intervention or implementation process will occur (i.e. Process); what resources may be necessary to achieve this (i.e. Provisions); and the underlying principles of the intervention (e.g. individual behavior change) and implementation process (e.g. building capacity for implementation) that will be scaled-up (i.e. Principles). Table 2 presents the descriptive criteria for the ‘five P’s’.

**Table 2:** Descriptive criteria of the Five P’s for effective implementation and scale up (Step 1)

The Five P’s	Descriptive criteria
1. People	The type and number of people that the intervention will reach, and the individuals that will be involved/required for implementation and scale-up
2. Place	The settings/organizations that will be involved/required for implementation and scale-up
3. Process	The intervention or implementation process that will occur in practice
4. Provisions	The resources (e.g. human, physical and fiscal) that will be necessary to achieve intervention implementation and scale-up
5. Principles	The underlying principles of the intervention (e.g. individual behavior change) and implementation process (e.g. building capacity for implementation) that will be scaled-up in practice

Outcomes from the five P’s inform: (i) actions required to make the intervention implementable and/or scalable; (ii) potential barriers to effective implementation and scale-up; and (iii) formative evaluation strategies and/or process measures that may improve implementation and scale-up. Researchers may complete the checklist independently or in collaboration with stakeholders identified in *Step 2*, however responses should be presented to stakeholders for validation or refinement.

### Step 2: Identify and engage key stakeholders across multiple levels within the delivery system(s)

**Fig. 2:**
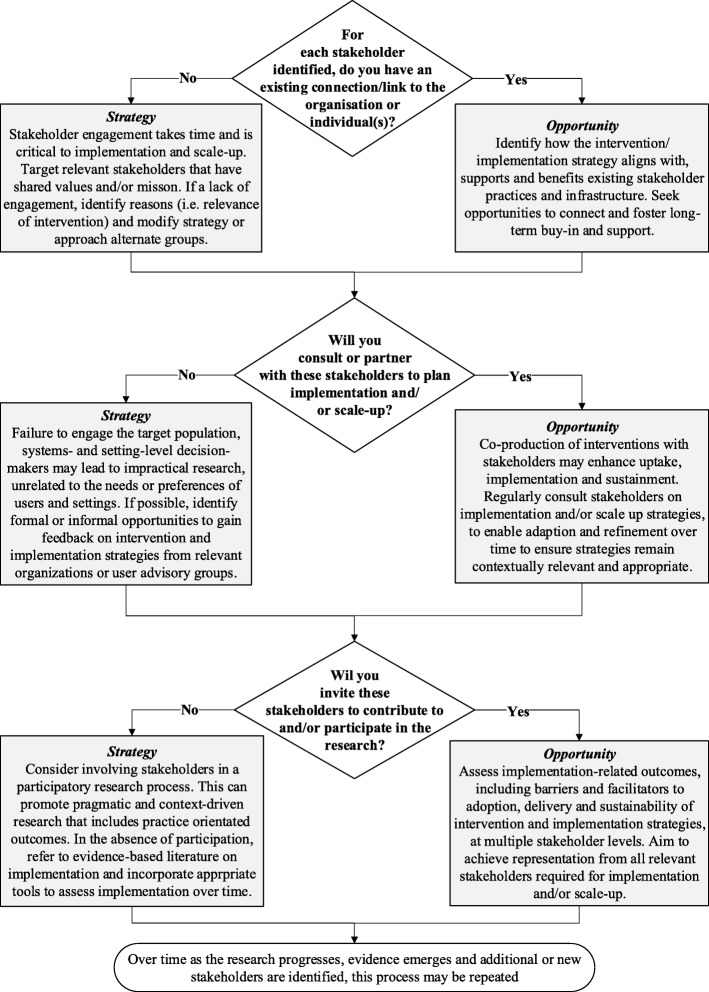
Decision tree to identify key stakeholders at multiple levels (Step 2)

The aim of *Step 2* is to identify and engage with key stakeholders (individuals or organizations) in interventions. Irrespective of the target setting, participatory research with stakeholders can facilitate implementation and sustainability of physical activity interventions, for example in community [39] and school settings [40], and scale up of physical activity interventions requires partnerships beyond the health sector [2]. An intervention that considers stakeholder priorities may lead to improved engagement and involvement [41], as implementation will be optimized through an understanding of an intervention’s likely or potential ‘fit’. Figure 2 presents a decision tree to facilitate stakeholder identification.

To operationalize the decision tree (Fig. 2), firstly identify stakeholders (individuals or organizations) that will: (i) fund and/or have overarching responsibility or ‘ownership’ for the intervention; (ii) disseminate (distribute) the intervention to the target setting and population; (iii) host and/or deliver the intervention either in the target setting or to the target population; and (iv) receive the intervention (target user). Stakeholder discussions should aim to increase awareness of the research, practice and policy activities involved in implementation and scale up, and facilitate effective information exchange, collaboration and use of existing knowledge [42]. Strategies for effective stakeholder engagement and communication should feature throughout implementation, to ensure transparency in roles and responsibilities, and agreement on outcome expectations. Depending on the context and scale of implementation (i.e. targeting 100 or 10,000 people), stakeholders may represent multiple distinct organizations or multiple distinct roles within any one organization. Depending on the type of intervention, stakeholders may also represent single or multiple ‘systems’ (i.e. health, education, transport systems).

Strategies and resources required to engage these stakeholders will therefore differ based on the type of intervention, scale of implementation and number of systems that will be involved in delivery. Outcomes from the decision tree will highlight opportunities to strengthen or seek new stakeholder relationships, and possible limitations if engagement and/or participation is suboptimal.

### Step 3: Identify contextual barriers and facilitators to implementation

**Fig. 3:**
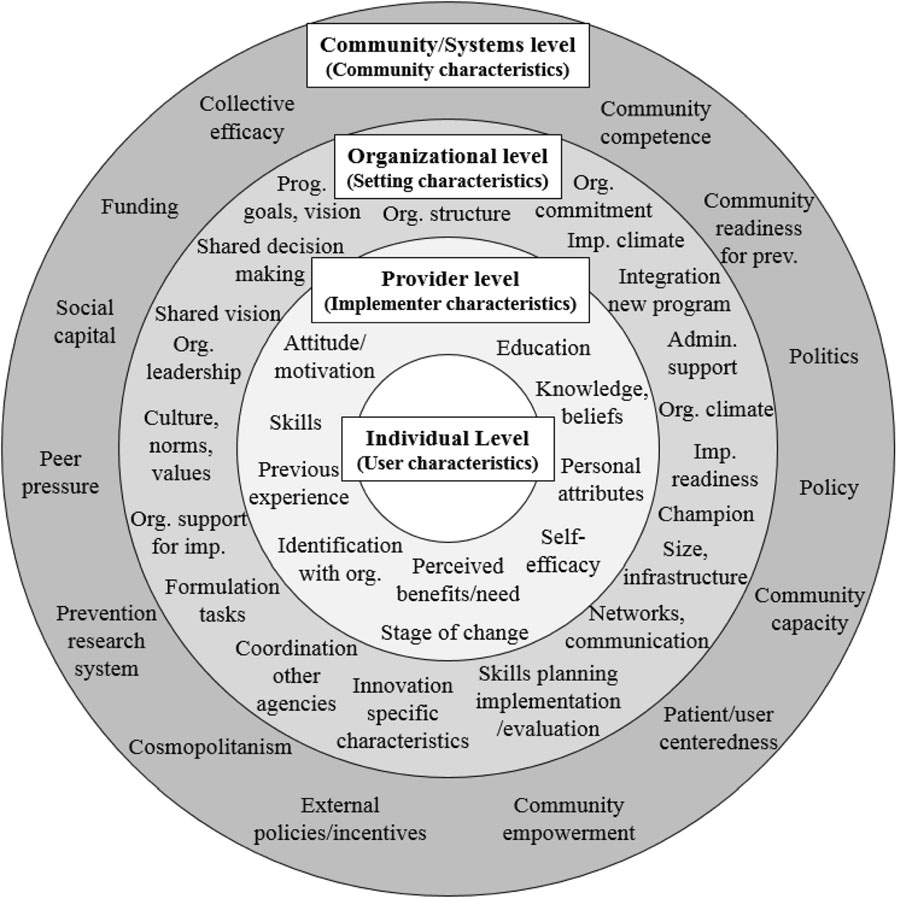
Ecological model of potential influences on intervention implementation in practice (Step 3)

The purpose of *Step 3* is to identify potential contextual barriers and facilitators to implementation and scale-up, to enhance integration of research findings into practice [32]. Figure 3 presents an ecological model depicting 45 of the 72 overlapping ‘common’ constructs, identified from the three frameworks. The remaining 27 ‘common’ constructs are not depicted in Fig. 3 for pragmatic reasons, as they either underpin another CFIR construct presented in Fig. 3 (see italicized constructs in Additional file 1) or are constructs that influence all levels of the ecological model. Constructs influencing all ecological levels include intervention characteristics (i.e. intervention’s legitimacy, quality/validity of evidence, adaptability, trialability, complexity, compatibility, relative advantage, design quality and cost) [14, 35], and the process of implementation (i.e. intervention specific and general capacity building [14, 17], and planning, engaging, executing, reflecting and evaluating the implementation process [35]).

The purpose of Fig. 3 (and Additional file 1) is to illustrate the breadth of commonly occurring factors that may require exploration with stakeholders during implementation planning and intervention testing. This compilation could be useful in pinpointing aspects of the intervention design and implementation/scale-up plan that may warrant refinement or further testing. The compilation can also be used as a reference tool when completing the 15-item checklist in *Step 1*, and provide a pictorial representation of potential challenges to implementation when engaging with stakeholders in *Step 2*.

### Step 4: Address potential barriers to effective implementation

The purpose of *Step 4* is to address potential barriers to effective implementation identified in *Step 3*, through refinements to the intervention and implementation process with stakeholders, and/or formal assessment of their impact on implementation and intervention outcomes through research testing. Table 3 presents example strategies to be incorporated during intervention and implementation planning (formative evaluation) and/or during intervention testing (process and outcome evaluation), which may improve research-practice translation.

**Table 3:** Example strategies to address multi-level contextual barriers to implementation (Step 4)

Level of identified barrier	Example strategies to address and/or assess contextual implementation barriers	
	*Formative evaluation strategies*	*Process evaluation measures*
Intervention population (Individual level)	• Focus groups with target population to explore barriers and facilitators to intervention design at scale and fostering sustained participation. • Needs assessments to address current gaps between what is available versus what is required by target population.	• Measure characteristics of responders and non-responders, and compare with general population to assess representativeness and generalizability. • Compare participant characteristics with retention and outcome variables. • Measure participant perceptions of intervention characteristics, and explore association with uptake, delivery and outcomes.
Implementers (Provider level)	• Focus groups with target implementers to explore existing implementation infrastructure and feasibility of intended intervention delivery. • Participatory approaches allow target user input on intervention design, training and implementation strategy, and can assist in identifying potential ‘champion’ implementers.	• Measure level of intervention delivery (i.e. dose delivered, dose received, and fidelity/adaptation) and assess associations with implementer characteristics. • Explore associations between level of delivery and intervention, and implementer characteristics. • Measure perceived barriers and facilitators to intervention delivery and sustainability, compare changes in delivery with outcomes over time.
Delivery setting/org. (Organizational level)	• Consultation/participatory research approaches with stakeholders (delivery settings who will support/provide the training/host implementers) on existing dissemination strategies and implementation infrastructure, to identify barriers to sustained implementation.	• Measure characteristics of delivery setting (i.e. size) and explore association with adoption decision, intervention delivery (i.e. dose) and intervention outcomes. • Compare characteristics of the delivery setting with perceived barriers and facilitators to adoption, intervention delivery and institutionalisation.
Community factors (Community/systems level)	• Consultation with organizations who will host/fund the intervention. Explore funding infrastructures, conflicting/supporting policies and local delivery context. • Partner with relevant stakeholders and users during intervention development, ensuring intervention addresses community priorities and applicable to context.	• Measure perceived contextual barriers to intervention adoption, delivery and sustainability. • Compare characteristics of participating communities with the real-world delivery context.

Translational formative evaluation [43] can include practice-based activities (i.e. strategic partnering with stakeholders, community members, decision-makers and opinion leaders associated with future implementation and scale-up), and research-based activities (i.e. needs assessments, feasibility and pilot trials) to assess local needs, values and intervention compatibility with the local context. Process measures are assessed in parallel to intervention testing, and can elucidate underlying mechanisms in the intervention context that may explain outcomes or how to move from research to practice [44]. Example ways of using formative and process evaluation to inform implementation and scale-up of physical activity interventions are described in the following section.

### Operationalizing the PRACTIS guide

To exemplify operationalization of the PRACTIS guide, we retrospectively applied the guide to four evidence-based physical activity interventions previously translated into clinical and community settings (see Additional file 2 for a summary of these case studies). To illustrate how outcomes from *Step 1* correspond to implementation planning, we present information from these example interventions against the criteria of the five P’s (Table 2). The example interventions have all been adopted and implemented at a state or national level in the United States or Australia and were designed with implementation in mind. These interventions were chosen as they adopted a range of intervention strategies and targeted varying populations and settings, for example: early childcare centers (Nutrition and Physical Activity Self-Assessment for Child Care [NAP SACC] [45]), the school setting (the Child and Adolescent Trial for Cardiovascular Health [CATCH] [46, 47]), and community-based/clinical healthcare contexts (Healthy Living after Cancer [HLaC] [48] and Move More [49]). Findings from earlier trials of NAP SACC have led to the development of an online delivery version to extend the program’s reach (‘Go NAP SACC’ [50]). For the purpose of this paper, we only draw on early publications detailing original study/intervention design and implementation planning processes.

The use of case studies to illustrate application of the guide highlights the variation in coverage of the four *Steps*. Consistent across all intervention examples, was that information gathered on the ‘five P’s’ to determine the parameters of the implementation context was used in each of the subsequent *Steps.* Each intervention adopted a participatory and collaborative approach to the co-production of evidence, drawing on stakeholders to guide the research design and implementation planning process, in addition to having input into intervention materials and resources. While these case studies are only briefly presented here and based on the available published descriptions of implementation planning, they highlight the strengths of a comprehensive, multilevel exploration of barriers and resolutions, and inclusion of formative and process evaluation to plan for implementation and scale-up.

## Discussion

Challenges and recommendations to improve intervention dissemination and implementation are well documented [31, 34, 51]. What is less well established is how researchers should apply this knowledge pragmatically to improve the implementation of physical activity interventions in real-world settings. The PRACTIS guide addresses this gap and demonstrates how to map features of the implementation setting, identify and engage important stakeholders, and anticipate and address potential barriers and facilitators to effective implementation and scale up.

It is assumed that interventions must first demonstrate efficacy under controlled conditions in a randomised trial, then effectiveness under real-world conditions, and finally evidence for potential scale up and subsequent dissemination [52]. In the context of behavioral interventions in particular, this sequential progression from efficacy to dissemination has been criticised on the basis that efficacy trials tell us little about the generalizability of interventions [53–55]. There is also the implicit assumption that dissemination and implementation may only be considered later in the research process. Contrary to Flay et al. (2005), we propose that conceptual and methodological issues in translation, constructs identified in implementation frameworks, and strategies to improve the translation of physical activity interventions, can and should be considered from the beginning of intervention development. Starting with the end application in mind can strengthen translation efforts [34], address challenges of dissemination [16], and may be linked to more successful scale-up efforts [56]. Engaging practice professionals and other stakeholders early in intervention planning may also strengthen research translation efforts and enhance application of the proposed steps [15].

The guide is also applicable when planning scaled-up interventions in other areas of public health prevention. Unlike discrete intervention trials targeting small populations, at scale the impact of system-level factors such as bureaucratic cultures, political environments and global trends may be amplified [57]. Funding and resources are also more likely to be sourced from within user organizations or the broader delivery systems (i.e. health, education, political), until intervention costs become integrated into national and local budgets [58]. Intervention flexibility and contextual adaptation are critical credentials for the widespread adoption of interventions across diverse sociocultural settings [56]. Irrespective of the scale of implementation, adaptation can be both desirable (such as adapted intervention modality to increase reach) and undesirable (such as a lower dose of delivery due to conflicting demands) [59]. Ascertaining intervention flexibility and associated effectiveness following contextual adaptation, should feature within all public health intervention planning. Successful scale up may depend on the interaction between an intervention’s intensity and the resources available within the delivery setting [5, 60]. The PRACTIS guide is hypothesized to result in intervention and implementation adaptations that align more closely to resources available within the delivery context.

Real-world implementation is an evolving process that mirrors constant changes in the physical and social environment. As the intervention and research progresses, and new information emerges, this guide may need to be revisited. Addressing all potential barriers to implementation (*Step 3*) may neither be feasible nor necessary, and decisions in *Step 4* are likely to be informed by barrier implications, researcher capacity and pragmatism. For example, system-level barriers such as existing policies which conflict with intervention objectives or a political system that does not prioritize preventive health, will require complex resolutions that are not immediately ‘solvable’. Researcher capability to respond to a barrier may therefore be limited, irrespective of well-meaning intentions or strong supportive evidence.

Abandonment (or de-implementation) of health interventions should occur, for example, when evidence contradicts existing practices [61]. However, de-implementation can depend on multiple factors, beyond scientific evidence, such as cultural, societal values and inertia [61]. For example, interventions that are ‘designed for scalability’ at an individual level (e.g. using an online modality to facilitate broad reach among target users) or have demonstrated evidence for effectiveness, may still fail to achieve sustained stakeholder support for implementation, due to entrenched organizational or system level factors. We hypothesize that de-implementation is best achieved when alternative options are available and a participatory approach is used to match intervention strategies to organizational resources as described in the PRACTIS guide. Following the guide may also help identify circumstances when further testing and/or adaptation of interventions should cease, or when abandon of implementation is more viable.

This guide is not without limitations and the complexities and costs of implementation within single and multiple systems should not be understated. To date, the majority of implementation research has emanated from clinical and health service contexts and it is unclear if such evidence is transferable to physical activity intervention research where the ‘place’ of delivery extends to a wide variety of community settings. Solutions to implementation barriers are also likely to differ by settings, multicomponent implementation strategies may be suited to different stages of implementation [30], and the appropriateness and effectiveness of strategies may vary by translation phase [62]. It is also less clear whether implementation variables should be prioritized in specific contexts, and which implementation strategy is best under which circumstance. Future research which investigates these core issues is needed. We encourage researchers to apply this population-focused guide and critically evaluate the proposed process.

## Conclusions

Complex characteristics of real-world implementation settings and the multilevel barriers to effective implementation are two of the many challenges faced when translating evidence-based interventions into practice. Those who are interested in translating physical activity promotion research into sustained practice are encouraged to use, and may benefit from, the PRACTIS guide to inform and operationalize their work. This guide is also relevant for clinical and public health researchers in other areas of prevention, to translate evidence-based interventions into real-world settings.

## Additional files

Additional file 1: Overlapping framework constructs. Table presenting constructs from the three implementation frameworks and corresponding overlap in constructs between frameworks. (DOCX 21 kb)

Additional file 2: Example operationalization. Table summary of four physical activity intervention illustrating operationalization of the PRACTIS guide (DOCX 25 kb)

## Abbreviations

CFIR: Consolidated framework for implementation research
ISF: Interactive systems framework
PA: Physical activity
PE: Physical education
PRACTIS: PRACTical planning for Implementation and scale-up
