# Supplementary material for: Implementation and scale up of population physical activity interventions for clinical and community settings: the PRACTIS guide
Source: Int J Behav Nutr Phys Act. 2018 Jun 8;15:51. doi: 10.1186/s12966-018-0678-0 (PMC5994105; doi:10.1186/s12966-018-0678-0)
Supplement: Supplementary file 1 — Overlapping framework constructs. Table presenting constructs from the three implementation frameworks and corresponding overlap in constructs between frameworks. (DOCX 21 kb) [file 12966_2018_678_MOESM1_ESM.docx]

Additional file 1. Overlapping framework constructs

| **Level** | **Overlapping ‘common’ framework constructs**  (Total N=72) | **Distribution of individual framework constructs**  (Total N=85) | | |
| --- | --- | --- | --- | --- |
|  |  | **ISF**  (N=23) | **CFIR**  (N=39) | **Ecological**  (N=23) |
| **Implementer characteristics (Provider level)** | 1. Education | X |  |  |
|  | 1. Previous experience with same/similar innovation | X |  |  |
|  | 1. Knowledge, Knowledge/beliefs regarding intervention | X | X |  |
|  | 1. Attitude towards innovation (innovation specific capacity) | X |  |  |
|  | 1. Motivation towards innovation (innovation specific capacity) | X |  |  |
|  | 1. Skills/Skill proficiency (innovation specific capacity) | X |  | X |
|  | 1. Perceived need for innovation (perceived local relevance) |  |  | X |
|  | 1. Perceived benefits of innovation |  |  | X |
|  | 1. Self-efficacy |  | X | X |
|  | 1. Individual stage of change |  | X |  |
|  | 1. Individual identification with organization |  | X |  |
|  | 1. Other general personal attributes (e.g. motivation, values, competence, capacity) |  | X |  |
| **Delivery setting/ organizational characteristics (Organizational level)** | 1. Organizational leadership (e.g. staffing considerations, setting priorities) | X |  | X |
|  | 1. Program goals/vision, | X |  |  |
|  | 1. Organizational commitment, shared vision/commitment (org. capacity general) | X |  | X |
|  | 1. Structural characteristics, size/Infrastructure (age), resources | X | X |  |
|  | 1. Skills for planning, implementation and evaluation | X |  |  |
|  | 1. Organizational climate/Positive work climate | X |  | X |
|  | 1. Organizational structure, decision making structure | X |  |  |
|  | 1. Innovation-specific characteristics (e.g. access to information about innovation) | X |  |  |
|  | 1. Networks and communications |  | X | X |
|  | 1. Culture (norms, values, basic assumptions), Organizational norms regarding change |  | X | X |
|  | 1. Implementation climate |  | X |  |
|  | 1. *Tension for change* |  | X |  |
|  | 1. *Relative priority* |  | X |  |
|  | 1. *Organizational incentives/rewards* |  | X |  |
|  | 1. *Goals and feedback* |  | X |  |
|  | 1. *Learning climate* |  | X |  |
|  | 1. Readiness for implementation |  | X |  |
|  | 1. *Leadership engagement* |  | X |  |
|  | 1. *Available resources* |  | X |  |
|  | 1. *Access to knowledge and information* |  | X |  |
|  | 1. Organizational support for implementation | X |  |  |
|  | 1. Integration of new programming (extent new innovation can be incorporated) |  |  | X |
|  | 1. Shared decision making (e.g. community participation/involvement, collaboration) |  |  | X |
|  | 1. Coordination with other agencies |  |  | X |
|  | 1. Formulation of tasks (e.g. workgroups, human resource management) |  |  | X |
|  | 1. *Champion (e.g. internal advocate) |  | X | X |
|  | 1. Managerial/administrative support |  |  | X |
| **Community characteristics**  **(Community/ systems level)** | 1. Community capacity | X |  |  |
|  | 1. Community readiness for prevention | X |  |  |
|  | 1. Community competency | X |  |  |
|  | 1. Community empowerment | X |  |  |
|  | 1. Collective efficacy | X |  |  |
|  | 1. Social capital | X |  |  |
|  | 1. Extent of patient/user-centeredness |  | X |  |
|  | 1. Cosmopolitanism (degree of external networks outside organization) |  | X |  |
|  | 1. Peer pressure (outside entity organization as affinity/competition with) |  | X |  |
|  | 1. External policies/incentives (policies, regulations) |  | X |  |
|  | 1. Prevention research system |  |  | X |
|  | 1. Politics |  |  | X |
|  | 1. Funding |  |  | X |
|  | 1. Policy |  |  | X |
| ****Intervention characteristics** | 1. Intervention source (legitimacy) |  | X |  |
|  | 1. Evidence strength and quality (validity of evidence) |  | X |  |
|  | 1. Relative advantage (perceptions of advantages of implementation) |  | X |  |
|  | 1. Adaptability (tailored, refined, reinvented), program modification/reinvention |  | X | X |
|  | 1. Trialability (ability to test an intervention on a small scale first) |  | X |  |
|  | 1. Complexity |  | X |  |
|  | 1. Design quality and packaging |  | X |  |
|  | 1. Cost |  | X |  |
|  | 1. *Compatibility (contextual fit, appropriateness) |  | X | X |
| ****Process of implementation** | 1. Planning |  | X |  |
|  | 1. Engaging (involving appropriate individuals in implementation & intervention use) |  | X |  |
|  | 1. *Opinion leaders* |  | X |  |
|  | 1. *Formally appointed internal implementation leaders* |  | X |  |
|  | 1. *External change agents* |  | X |  |
|  | 1. Executing (individuals in organization with influence on implementation) |  | X |  |
|  | 1. Reflecting and evaluating (before, during, after implementation) |  | X |  |
|  | 1. Innovation-specific capacity building (providing information, training), Training | X |  | X |
|  | 1. Technical assistance |  |  | X |
|  | 1. General capacity-building (enhance infrastructure, organizational motivation) | X |  |  |

ISF=Interactive Systems Framework, CFIR=Consolidated Framework for Implementation Research, Ecological=Ecological framework for effective implementation. *CFIR construct ‘compatibility’ located under ‘implementation climate’, and CFIR construct ‘champion’ located under ‘engaging’ at [www.cfirguide.org](http://www.cfirguide.org) (accessed 10.01.18). **Constructs and italicized CFIR constructs (no’s 24-28, 30-32, 65-67) not represented in Figure 3 Ecological Model.
