# Supplementary material for: Implementation and scale up of population physical activity interventions for clinical and community settings: the PRACTIS guide
Source: Int J Behav Nutr Phys Act. 2018 Jun 8;15:51. doi: 10.1186/s12966-018-0678-0 (PMC5994105; doi:10.1186/s12966-018-0678-0)
Supplement: Supplementary file 2 — Example operationalization. Table summary of four physical activity intervention illustrating operationalization of the PRACTIS guide (DOCX 25 kb) [file 12966_2018_678_MOESM2_ESM.docx]

Additional file 2. Example operationalization of the PRACTIS guide

|  | **Intervention example** | | | |
| --- | --- | --- | --- | --- |
|  | **Nutrition and Physical Activity Self-Assessment for Child Care (NAP SACC)** | **Child & Adolescent Trial for Cardiovascular Health (CATCH)** | **Healthy Living after Cancer (HLaC)** | **Move More** |
| *Target outcome*  *Population*  *Setting* | *PA, diet*  *Children (3-5yrs)*  *Early childcare centers* | *PA, diet, smoking abstention*  *Children (7-11yrs)*  *School setting* | *PA, diet, weight loss*  *Adults (18+yrs)*  *Community-based, clinical* | *PA*  *Adults (18+yrs)*  *Clinical setting* |
| **Step 1: Characterize implementation setting parameters** | **Place**: Early childcare centers (ECC).  **People** & **Process**: 6month intervention targeting ECC directors and staff. Research team trained technical assistance providers (via 3x workshops) as NAP SACC consultants, who then recruited ECC. Centers completed the self-assessment instrument, evaluated changes and developed action plans with consultants. Consultants led continuing education workshops, provided ongoing support for implementation (in-person visits, phone calls)  **Provisions**: Self-assessment instrument, continuing educational workshop (and materials), action plans, toolkit of technical assistance materials, resource manual.  **Principles**: *Intervention* – targets individual and environmental change based on SCT. *Implementation* – Uses existing infrastructure of public health professionals by expanding staff roles, and uses self-assessment for assessment/monitoring so center-directed change. | **Place**: School with home-based components.  **People** & **Process**: School Principals, food service managers, students and families facilitated or received the intervention. Delivered by teachers/PE specialists and service staff. CATCH staff trained implementers on site, providing regular support visits. Teachers recruited families with flyers.  **Provisions**: Recruitment flyers, weekly curricula, activity packets, structured games/activities, participation certificates, and incentives  **Principles**: *Intervention* –  Psychosocial risk factors assoc. with unhealthy eating, physical inactivity, and smoking. Changes in the curricula reinforced at home. *Implementation* – Targets school-wide via principles associated with adoption (e.g. relative advantage), implementation (e.g. training to enhance fidelity), and institutionalization (e.g. early collaboration). | **Place**: Telephone-delivered via helplines of state-based Cancer Councils (CC).  **People** & **Process**: 6month intervention targeting cancer survivors. Delivered by trained CC nurses/allied health prof.; led by a lead CC nurse. CC reps trained via 2 day workshop, followed by train-the-trainer. CC reps promote HLaC on site, delivering weekly, fortnightly monthly calls (max. 12), with weekly debrief to lead CC nurse. Research team provided email and monthly case management support.  **Provisions**: Training manual and 2-day training workshop, phone call protocol and participant workbook.  **Principles**: *Intervention* - developing participant skills in goal setting, self-monitoring, problem solving, identifying social support, stimulus control, positive self-talk and self-reward.  *Implementation* – Used existing service delivery model, built capacity among CC for delivery and staff-led evaluation. | **Place**: Healthcare clinics.  **People** & **Process**: Participants recruited via Family Practice physician referral. Physicians engaged in developing patient referral scheme with research team. Health educators delivered group sessions in clinics. Intervention involved 2 x 2hr group visits over 2 months with a follow-up support call 8wks post second group visit.  **Provisions**: Educational handouts, personal action plans, interactive group activities, attendance rewards.  **Principles**: *Intervention* - group dynamic principles, which includes targeting changes in group structure, environment and processes  *Implementation* – Used an integrated research-practice partnership within an existing care delivery system to increase system-wide scale-up. |
| **Step 2:**  **Identify and engage key stakeholders** | Stakeholders represented the provider, organizational and community/systems levels. Created an advisory group (child health professionals, ECC providers, and a county extension agent). | Stakeholders represented provider, organizational and community/systems levels. School district collaboration and/or participation was established from the outset, which informed planning institutionalization, sustaining commitment to CATCH objectives, and modifications over time. | Engaged policy, practice and service delivery stakeholders, clinicians, and consumer advocates to guide study design, evaluation and intervention protocol adaptation, and oversee implementation to guide advocacy for sustained funding. | System-level integrated research-practice partnership with the Kaiser Permanente Colorado healthcare system. Community and setting level stakeholders: Chief and Director of Preventive Medicine, the Director of Health Education, health educators, and the Physician in Charge and Medical Office Administrator. |
| **Step 3:**  **Identify contextual barriers and facilitators** | Org. and provider level barriers: i) adoption of NAP SACC among ECC, and uptake and ongoing support for implementation among ECC staff, ii) implementation capacity (e.g. NAP SACC consultants requiring support beyond the scope of training), iii) challenges to effective (quality) implementation of NAP SACC over time, and iv) implementation and outcome sustainability. | Org. and provider level barriers: i) adoption (e.g. school perceived need, relative advantage and compatibility); ii) effective delivery (e.g. low reach, teacher fidelity); iii) institutionalization (e.g. low school ‘ownership’ and parental support); iv) school structure (e.g. food service staff turnover); v) CATCH staff characteristics (e.g. skills); vi) system-level policies and; vii) implementer and recipient characteristics (e.g. education, knowledge), | Org. and provider level barriers: i) capacity of CC staff to implement and evaluate HLaC, and ii) ensuring embeddedness and contextual fit with CC. At a systems level: i) sustained funding beyond research trial. | Org. level barriers: i) stakeholders perceived potential burdensomeness (intensity and practicalities for a clinical setting). Barriers associated with intervention characteristics: i) perceived relative advantage and sustainability of effects. |
| **Step 4: Address/assess barriers** | *Formative evaluation*: Stakeholder advisory group provided feedback to guide development of intervention processes and materials. Interviews conducted with providers, directors/assistant directors, teachers, and foodservice staff. Focus groups with parents to obtain provider and parent views of nutrition and physical activity environments and regulatory and rating systems in ECC.  *Strategies to address barriers*: Incentivized training workshops with continuing education (CE) credits. A Personal Health and Wellness workshop added to address staff health interests. NAP SACC resource manual to overcome potential implementation barriers. Used Center-directed assessments to increase sustainability of change.  *Process/outcome evaluation*: Pilot study led to revisions to self-assessment instrument. | *Formative evaluation*: Pragmatic challenges to dissemination, adoption, delivery and institutionalization were integrated into a causal model underpinning CATCH implementation. Strategies to overcome adoption included social incentives, and heightened awareness of CATCH values and benefits to teachers and children. Clear/user friendly training manuals were developed, and training emphasised completeness, involved active role modelling/ rehearsal, and feedback strengthened effective delivery.  *Process evaluation:* Documented recruitment methods, barriers to adoption, and teacher commitment level to CATCH components explained outcomes and informed dissemination. Interviews, student reports, class observations and participation rates informed on CATCH delivery and participant exposure. | *Formative evaluation*: Concept development workshop to engage policy and practice stakeholders, and plan three phases of HLaC (1. knowledge transfer and capacity building; 2. implementation and evaluation; 3. funding advocacy).  *Strategies to address barriers*: In partnership, CC developed HLaC referral pathway and emphasised ‘fit’ of HLaC with CC strategic planning. HLaC embedded in existing CC service delivery model, used existing CC resources, and aligned with organizational mission/ priorities. Built capacity of CC staff to self-monitor and evaluate – used for wider communication and advocacy for sustained funds.  *Process/outcome evaluation*: Evaluation of both service-led indicators, costs and cancer survivor outcomes. | *Formative evaluation*:  Strategies: Frequency & time of group-based contact was greatly reduced. Behavioral target changed from group exercise to exercise on one’s own with a specific focus on continued increases in physical activity even after the program was complete. |

Authors: Nutrition and Physical Activity Self-Assessment for Child Care (NAP SACC) (Ammerman, et al., 2007), the Child and Adolescent Trial for Cardiovascular Health (CATCH) (Perry et al., 1990, McGraw et al., 1994), Healthy Living after Cancer (HLaC) (Eakin et al. 2015), Move More (Estabrooks et al., 2011). PA - Physical Activity, PE - Physical Education, SCT - Social Cognitive Theory.
